# Psoas Abscess: A Possible Missed Diagnosis in the Emergency Department

**DOI:** 10.7759/cureus.101347

**Published:** 2026-01-12

**Authors:** José Alonso Silva Torres, Grace Thomas, Jonathan Van Dellen

**Affiliations:** 1 General Surgery and Urology, Croydon University Hospital, London, GBR; 2 Diabetes and Endocrinology, Croydon University Hospital, London, GBR; 3 General Surgery, Croydon University Hospital, London, GBR

**Keywords:** diagnosis delay, fever of unknown origin, hydronephrosis, inflammatory marker, pain in hip, retroperitoneal infection, secondary psoas abscess

## Abstract

A woman in her 70s was admitted to the Emergency Department with non-specific symptoms, such as fever, cough, dysuria, and hip pain with limited mobility, along with elevated inflammatory markers. She was initially diagnosed with a lower respiratory tract infection. However, she was readmitted to the hospital three days later due to worsening symptoms. The patient was eventually diagnosed with a secondary psoas abscess caused by underlying appendicitis and was successfully treated. This case highlights the diagnostic challenge of psoas abscess and underscores the importance of considering this uncommon condition in patients presenting with hip pain, fever and elevated inflammatory markers.

## Introduction

The psoas muscle is located bilaterally in the posterior abdominal wall. It has a proximal attachment along the lateral borders of the vertebral bodies from the twelfth thoracic (T12) to the fifth lumbar (L5) vertebrae and a distal attachment at the lesser trochanter of the femur. Together with the iliacus muscle, it forms the iliopsoas, which is the primary flexor of the hip. The psoas muscle lies within the retroperitoneal space, where several venous plexuses are found in close proximity, a feature that may explain its predisposition to the development of infections [1]. Closely related to this muscle on the right side are several important anatomical structures, including the appendix, cecum, regional lymph nodes, and inferior vena cava [2].

A psoas or iliopsoas abscess is a rare condition characterised by a localised collection of pus within the iliopsoas compartment. A study conducted in the UK in 1980 reported an annual incidence of psoas abscess of 0.4 cases per 100,000 people [3], although its true incidence remains uncertain [4]. Psoas abscesses can develop as a result of various infectious sources and may lead to serious complications such as paralytic ileus, hydronephrosis, and septic arthritis of the hip [5]. However, the lack of specific clinical manifestations makes diagnosis and timely treatment challenging, and if left untreated, this condition can be life-threatening, with reported mortality rates of up to 20% [6]. Computed tomography is considered the imaging modality of choice for the diagnosis of this condition [4].

Herein, we present a case of a secondary psoas abscess caused by underlying atypical appendicitis, emphasising the importance of early diagnosis and appropriate imaging and treatment.

## Case presentation

A woman in her late 70s was admitted to the Emergency Department with fever, tachycardia, cough, dysuria, and worsening right-sided hip pain for several days. Her past medical history included bilateral hip osteoarthritis, chronic obstructive pulmonary disease, and a radical hysterectomy for cervical cancer conducted 17 years before. In the initial clinical history, the patient denied any chest or abdominal pain, as well as nausea or vomiting at that time. However, she reported having experienced a few days of lower abdominal pain and nausea two weeks earlier, which resolved spontaneously after taking only over-the-counter analgesics. The patient also stated that she did not have any regular medication. Physical examination revealed decreased air entry on the right side of the chest, while abdominal palpation was unremarkable. The patient did not have hip tenderness on palpation. Vital signs confirmed tachycardia at 105 beats per minute (normal heart rate 60-100 bpm), hypotension 104/48 mmHg (normal systolic blood pressure 90-120, normal diastolic blood pressure 60-80 mmHg), and fever of 38.1 °C (normal temperature: 36-37.5 °C) (Table 1).

**Table 1:** Vital signs during first Emergency Department attendance and subsequent admission

Vital signs	1st Emergency Department presentation (Day 0)	Second Emergency Department presentation and admission (3 days later)	Admission Day 2 - Interventional radiologist drainage (5 days later)	Admission Day 3 – 24 hours post-interventional radiologist drainage (6 days later)	Admission Day 7 - Discharge day (10 days later)	Reference range
Systolic blood pressure	104 mmHg	104 mmHg	118 mmHg	112 mmHg	112 mmHg	91 – 120 (mmHg)
Diastolic blood pressure	48 mmHg	61 mmHg	57 mmHg	60 mmHg	70 mmHg	60 – 80 (mmHg)
Mean arterial pressure	67 mmHg	78 mmHg	84 mmHg	90 mmHg	84 mmHg	70 - 100 (mmHg)
Heart rate	105 beats/min	103 beats/min	79 beats/min	78 beats/min	87 beats/min	60 – 100 (beats/min)
Respiratory rate	15 breaths/min	18 breaths/min	16 breaths/min	18 breaths/min	16 breaths/min	12 – 20 (breaths/min)
Oxygen saturation	95% (room air)	96% (room air)	96 % (room air)	95 % (room air)	95 % (room air)	≥94 %
Temperature	38.1 °C	37.6 °C	36.7 °C	36.5 °C	36.8 °C	36.0 – 37.5 (°C)

Blood tests conducted during the first Emergency Department presentation revealed an inflammatory response with elevated white cell count and neutrophilia (Table 2). Renal function tests indicated an estimated glomerular filtration rate below normal ranges, while C-reactive protein levels were elevated. The urine dipstick test and chest X-ray did not show any features of infection. A urine culture was not sent to the laboratory. Based on the available test results and clinical examination, the patient was diagnosed with a possible lower respiratory tract infection. The patient was discharged home with a prescription for oral broad-spectrum antibiotic, amoxicillin-clavulanic acid 625 mg, to be taken three times daily for seven days. A follow-up review with the community medical team was arranged two days after discharge from the Emergency Department.

**Table 2:** Laboratory results

Laboratory value	First Emergency Department presentation (Day 0)	Second Emergency Department presentation and admission day (3 days later)	24 h after interventional radiologist drainage and antibiotic treatment ( 6 days later)	Admission Day 7 - Discharge day (10 days later)	Reference value
White cell count	13.0 × 10⁹/L	20.6 × 10⁹/L	12.2 × 10⁹/L	9.4 × 10⁹/L	4.0–11.0 × 10⁹/L
Neutrophils	10.9 × 10⁹/L	18.0 × 10⁹/L	10.2× 10⁹/L	6.4 × 10⁹/L	1.5–8.0 × 10⁹/L
Estimated glomerular filtration rate	85 mL/min/1.73m2	57 mL/min/1.73m2	>90 mL/min/1.73m2	88 mL/min/1.73m2	90 – 120 mL/min/1.73m2
C-reactive protein	224 mg/L	419 mg/L	187 mg/L	5.0 mg/L	0-5 mg/L

**Figure 1:**
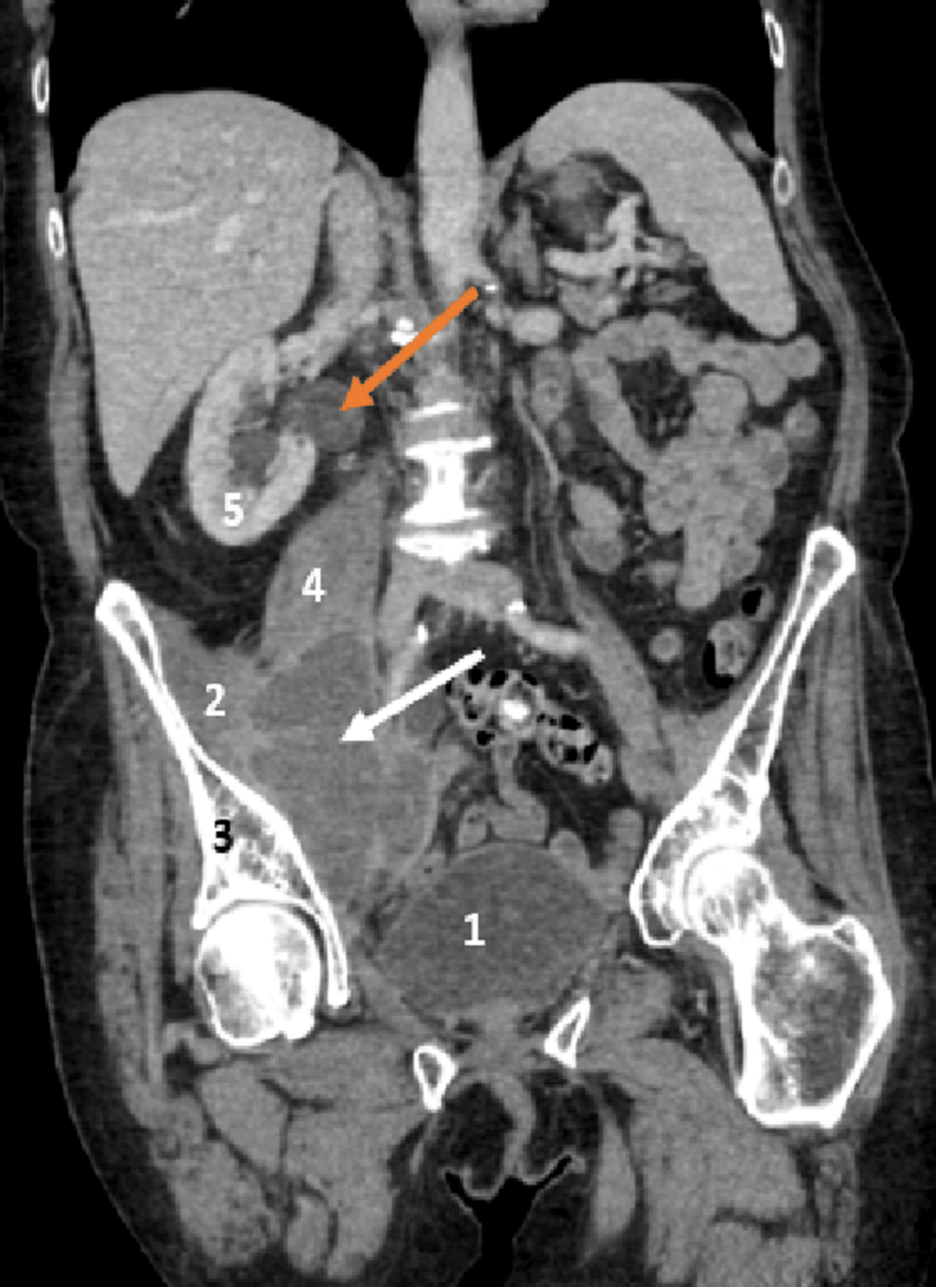
Contrast-enhanced computed tomography abdomen and pelvis coronal view 1. Bladder, 2. Iliacus muscle, 3. Pelvis, 4. Psoas muscle, 5. Right kidney White arrow: Psoas abscess; Orange arrow: Hydroureteronephrosis

**Figure 2:**
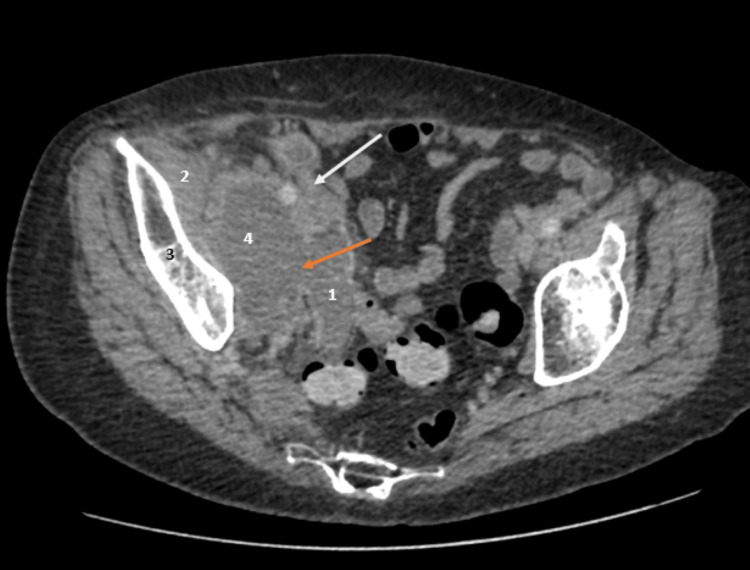
Contrast-enhanced computed tomography abdomen and pelvis axial view 1. Dilated appendix, 2. Iliacus muscle, 3. Pelvis, 4. Psoas abscess White arrow: Base of appendix, Orange arrow: Ruptured appendix communicating with abscess

The patient returned via ambulance to the Emergency Department three days after discharge due to a worsening clinical status despite ongoing antibiotic treatment. On examination, the notable finding was suprapubic and right iliac fossa tenderness, which had not been present during previous attendance. Blood tests revealed an increase in white cell count and C-reactive protein levels (Table 2). Throughout the patient's admission, intravenous antibiotics and analgesia were prescribed. A computed tomography scan of the abdomen and pelvis with contrast was done, and it revealed a 77 × 55 mm axial and 90 mm craniocaudal iliopsoas abscess, which was causing hydroureteronephrosis (Figure 1). The base of the appendix was visible; however, the tip was not (Figure 2).

**Figure 3:**
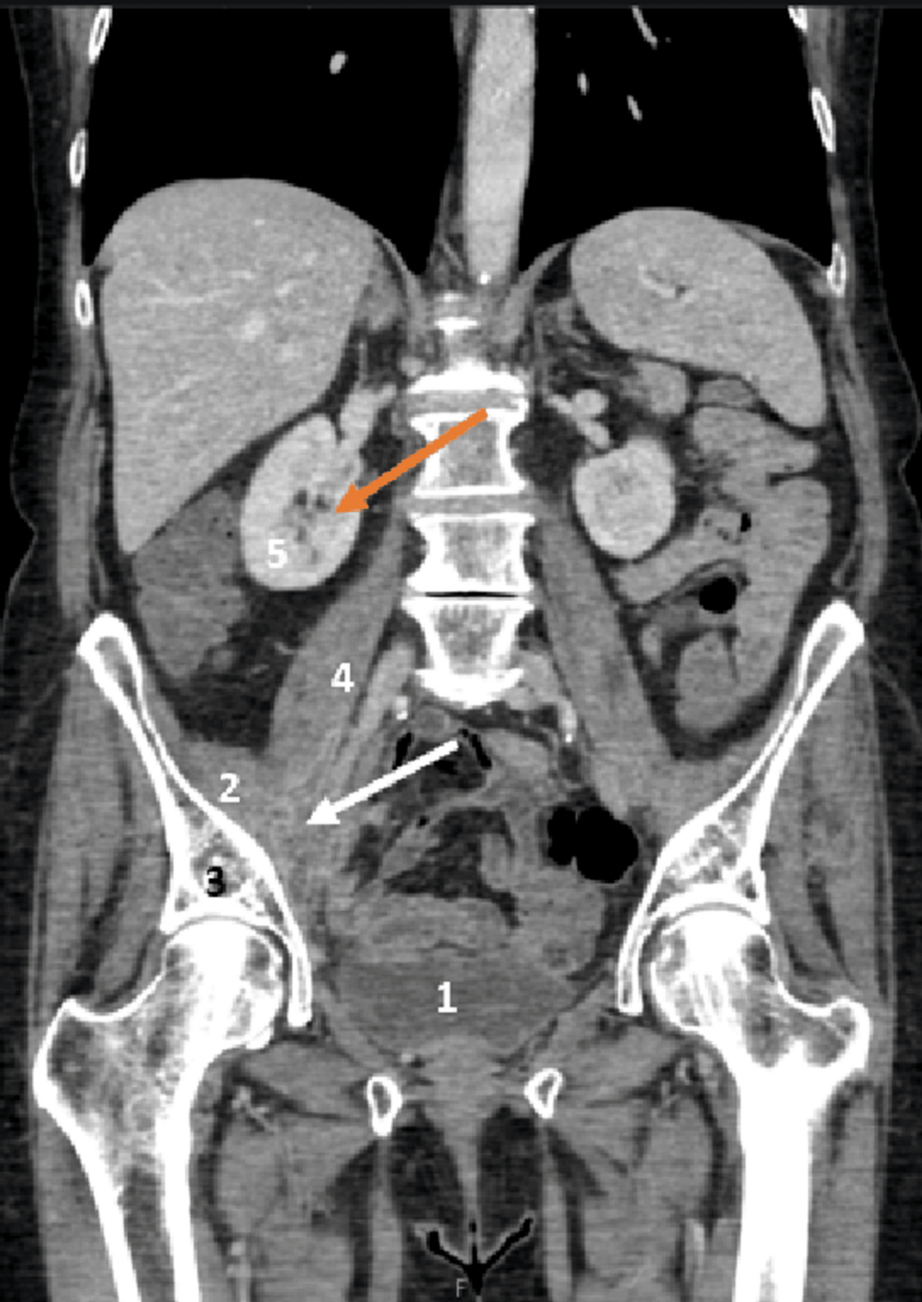
Contrast-enhanced computed tomography abdomen and pelvis coronal view after ultrasound-guided drainage of the psoas abscess 1. Bladder, 2. Iliacus muscle, 3. Pelvis, 4. Psoas muscle, 5. Right kidney White arrow: The psoas abscess decreased in size; Orange arrow: Resolved hydroureteronephrosis

Ultrasound-guided drainage of the fluid collection was performed two days after the second admission (Figure 3). This procedure was delayed because of a high workload in the Interventional Radiology department at our hospital. Immediate hip flexion and extension without pain were noted after approximately 150 mL of fluid had been drained. Pus sample analysis results revealed scanty growth of *Citrobacter freundii* (Gram-negative bacilli), moderate growth of *Streptococcus constellatus* (Gram-positive bacilli), and heavy growth of *Streptococcus intermedius *(Gram-positive bacilli) and anaerobes. The complete intravenous antibiotic course consisted of Ceftriaxone 2 g every 24 hours for 7 days, plus metronidazole 400 mg every 8 hours for 7 days, along with a single dose of piperacillin-tazobactam 4.5 g and a single dose of gentamicin 330 mg. The rationale for our antimicrobial regimen was based on local microbial epidemiology and culture results. Ceftriaxone was selected for broad Gram-negative and streptococcal coverage, while metronidazole was added to ensure adequate anaerobic coverage. The initial single doses of piperacillin-tazobactam and gentamicin were administered to provide prompt broad-spectrum coverage in the acute setting until culture results became available.

Treatment with antibiotics and drainage led to an improvement of inflammatory parameters within 24 hours (Table 2). Four days after the intervention, blood tests demonstrated normalisation of the white cell count and C-reactive protein levels. On the same day (day seven of admission), the patient was discharged home with a 6-day course of oral antibiotics consisting of amoxicillin-clavulanic acid 625 mg three times daily, ciprofloxacin 500 mg twice daily, and metronidazole 400 mg three times daily, in accordance with culture sensitivities, local antibiotic guidelines, and microbiology advice. The patient completed the prescribed antibiotic regimen and was scheduled for follow-up in the clinic eight weeks later. No further presentations to the Emergency Department were recorded.

## Discussion

The patient was ultimately diagnosed with a secondary psoas abscess arising from underlying appendiceal pathology. This was supported by a history of lower abdominal pain occurring two weeks prior to the initial Emergency Department presentation, which resolved spontaneously and may represent transient symptom improvement following appendiceal perforation. Further evidence included non-visualisation of the appendiceal tip on imaging and the polymicrobial nature of the pus obtained at drainage. Collectively, the clinical history, radiological findings, and microbiological results strongly suggest appendicitis as the primary source of the abscess. Diagnostic suspicion was delayed due to the initially nonspecific presentation and the absence of clinical features suggestive of appendicitis at the time of first assessment.

Primary psoas abscess occurs when a pathogen, frequently *Staphylococcus aureus*, spreads via lymphatic or haematogenous circulation from a distant location. In contrast, secondary psoas abscess is predominantly polymicrobial, resulting from the contiguous spread of pathogens from an adjacent infectious focus or muscle trauma. Secondary abscess can be triggered by underlying infections, including gastrointestinal (colitis, appendicitis), musculoskeletal (Pott's disease, septic arthritis, prosthetic joint infection), cardiovascular (endocarditis, infected aortic graft), genitourinary (pyelonephritis, cystitis), vascular (infected aortic aneurysm, acute mesenteric ischemia), and malignancies [7,8].

The clinical manifestations of psoas abscess are often nonspecific and may include weight loss, malaise, or flank pain [7]. Although the classic triad of fever, back pain, and limited hip movement can aid in the diagnosis, it is present in only about 30% of cases [9]. Therefore, relying on it alone may lead to underdiagnosis. However, a positive "psoas sign" or a palpable mass in the inguinal area may suggest the presence of this condition [7]. For accurate diagnosis, a detailed clinical history and thorough examination are essential, including laboratory tests, imaging studies, and blood cultures to identify the source of the infection.

During the clinical examination at the patient’s first presentation to the Emergency Department, she presented with a fever of unknown origin and other nonspecific symptoms, including cough and dysuria, which initially led the Emergency Department doctors to suspect both a lower respiratory tract infection and a lower urinary tract infection. The hip pain was presumed to be associated with the patient’s chronic hip osteoarthritis. Nevertheless, a more detailed assessment of the right hip, including a targeted evaluation of the psoas muscle with elicitation of the psoas sign, might have helped identify the psoas as the source of pain rather than the hip joint. Importantly, the patient did not report any abdominal pain at that time, and abdominal examination revealed a soft, non-tender abdomen. These factors contributed to a delay in establishing the correct diagnosis.

Laboratory investigations were also nonspecific, showing leucocytosis (13.0 × 10⁹/L) and markedly elevated C-reactive protein levels (204 mg/L), suggesting an underlying inflammatory process (Table 2), consistent with previous literature describing psoas abscess [8]. Although uncommon, lower urinary tract symptoms, such as dysuria, may result from extra-urinary causes [10]. In this case, the patient’s dysuria with a negative urine dipstick test was later attributed to compression of the right ureter by a psoas abscess, as demonstrated on computed tomography, which revealed hydroureteronephrosis. Additionally, given the winter season, the leucocytosis and elevated CRP, alongside the cough and a history of chronic obstructive pulmonary disease, the presentation was interpreted as a very likely lower respiratory tract infection.

The patient initially presented with tachycardia (105 bpm) and fever (38.1°C); however, her vital signs normalised following administration of intravenous antibiotics and 0.9% sodium chloride. Computed tomography is the gold standard for diagnosing a psoas abscess [11]. However, it was not performed during the first presentation due to a normal abdominal examination and absence of gastrointestinal symptoms. She was discharged with nonspecific symptoms, including fever, cough, and dysuria, along with elevated inflammatory markers, but outpatient follow-up was arranged by the community medical team. Although some may question why the patient was not admitted initially, her stable clinical condition following intravenous therapy supported the Emergency Department team’s decision for close outpatient monitoring rather than inpatient admission.

However, when the patient re-presented three days later following assessment by the community medical team, her clinical picture had evolved. She now reported suprapubic pain and left iliac fossa discomfort, accompanied by leucocytosis (20 × 10⁹/L) and markedly elevated C-reactive protein, nearly double the previous value, prompting consideration of a contrast-enhanced computed tomography scan. Contrast-enhanced computed tomography of the abdomen and pelvis confirmed a right-sided collection (Figure 1). During the procedure, the tip of the appendix was not visualised, raising the possibility of perforated appendicitis as the source of infection. The development of a secondary psoas abscess is frequently associated with appendicitis due to the anatomical proximity of the appendix to the psoas muscle [6]. This association is particularly relevant considering the high global incidence of acute appendicitis in adults annually [12]. Furthermore, studies have shown that gastrointestinal inflammatory conditions, including appendicitis, are directly associated with up to 30% of psoas abscess cases [13]. However, as reported in another case [14], our patient did not initially exhibit clinical signs suggestive of this pathology, which contributed to it being overlooked on physical examination and only identified through imaging. Computed tomography also revealed early hydroureteronephrosis secondary to ureteric compression by the psoas abscess, a complication previously described in similar cases [15,16].

Early treatment of psoas abscess is critical, as mortality rates can be high due to sepsis [17]. Abscesses that are relatively small (<3 cm) and simple are reported to respond better to antibiotic therapy alone. Abscesses larger than this, however, are generally managed with image-guided drainage in addition to broad-spectrum antibiotics to achieve optimal outcomes [13]. Percutaneous drainage is the preferred approach over surgical intervention, and it is typically guided under ultrasound or contrast-enhanced computed tomography. Surgical drainage is associated with significant morbidity, particularly in septic patients [18]. Therefore, it is indicated when the percutaneous drainage fails, the abscess is multiloculated, or there is an underlying intra-abdominal condition (e.g., diverticulitis or severe Crohn's disease) [10]. Ultrasound-guided drainage performed in our patient resulted in a successful outcome (Figure 2) with an immediate improvement in hip mobility and inflammatory indicators (Table 2). Pus cultures revealed polymicrobial growth, supporting the diagnosis of a secondary psoas abscess. The antibiotic course was completed according to our trust's antimicrobial guidelines. Still, follow-up was advised, as relapse has been reported in 15.8% of cases within the first year [8].

Despite initial difficulties in suspecting a secondary psoas abscess arising from underlying appendicitis, the patient in this case was successfully treated with drainage and antibiotic therapy guided by abscess culture results, local guidelines, and microbiology input. However, the presence of fever of unknown origin, dysuria, and right hip pain, combined with elevated inflammatory markers at the initial visit, should have prompted a more detailed assessment of the hip rather than attributing the pain solely to chronic osteoarthritis, as well as consideration of an earlier computed tomography scan to identify the underlying pathology. This case underscores the importance of including psoas abscess in the differential diagnosis of hip pain and fever. Increased awareness may facilitate earlier detection and reduce the risk of missed diagnoses, as well as the associated morbidity and mortality related to this condition or its underlying causes.

## Conclusions

Psoas abscess is an uncommon but possibly life-threatening condition, and failure to identify it promptly can result in significant morbidity and even death. Diagnosis is challenging because there are no pathognomonic symptoms, and the classic triad is present in less than half of patients. A careful assessment of the full clinical picture is therefore essential, ensuring that acute symptoms are not mistakenly attributed to chronic conditions. In cases where patients report hip pain, a focused examination of the psoas muscle, particularly eliciting the psoas sign, can provide valuable diagnostic clues. Computed tomography remains the gold standard imaging modality and should be obtained early when clinical suspicion exists. Furthermore, in patients presenting with dysuria and hip pain, a normal urine dipstick should prompt clinicians to broaden the differential diagnosis beyond urinary tract infection. In summary, these points highlight the importance of maintaining a high index of suspicion to enable timely diagnosis and effective management of psoas abscess.
